# Cutaneous Basal Cell Carcinoma In Situ: A Review of the World Literature

**DOI:** 10.7759/cureus.69691

**Published:** 2024-09-19

**Authors:** Philip R Cohen, Razelle Kurzrock

**Affiliations:** 1 Dermatology, University of California, Davis Medical Center, Sacramento, USA; 2 Dermatology, Touro University California College of Osteopathic Medicine, Vallejo, USA; 3 Maples Center for Forensic Medicine, University of Florida College of Medicine - Gainesville, Gainesville, USA; 4 Medicine, Medical College of Wisconsin Cancer Center and Genome Sciences and Precision Medicine Center, Milwaukee, USA; 5 Oncology, WIN Consortium, Villejuif, FRA; 6 Oncology, University of Nebraska Medical Center, Omaha, USA

**Keywords:** basal, cancer, carcinoma, cell, cutaneous, fibroepithelioma, in situ, invasive, molecular, skin

## Abstract

Cutaneous basal cell carcinoma (BCC) in situ is a recently recognized subtype of the skin neoplasm in which the abnormal cells are confined to the epidermis. BCC in situ of the skin was previously referred to as a superficial BCC. A review of the world literature has revealed 10 cutaneous BCCs in situ that have been described in nine patients but likely reflect a more general phenomenon. The neoplasm typically presents as an asymptomatic red plaque on the abdomen, upper extremity, back, and chest. Pathologic changes frequently show confluent tumor cells along the epidermal basal layer or superficial aggregates of neoplastic cells that are contiguous with the epidermis and extend into the dermis. Genomic evaluation has been performed in neoplasms from one individual with cutaneous BCC in situ and metastatic BCC; like other variants of BCC, an aberration of the *PTCH1* gene was observed. In contrast to his liver metastasis, the in situ carcinoma had a lower tumor mutational burden, lacked programmed death-ligand 1 *(PD-L1)* and programmed death-ligand 2 *(PD-L2)* amplification and had a distinct *PTCH1* mutation, suggesting that the in situ BCC of his skin and the metastatic BCC of his liver were derived from different clones of cells.

## Introduction and background

Basal cell carcinoma (BCC) is the most common skin cancer worldwide. BCC has been classified as a tumor with either a low risk of recurrence or metastasis (including those tumors with nonaggressive pathologic features, such as infundibulocystic with adnexal differentiation, fibroepithelial, nodular, pigmented, and superficial) or a higher risk of recurrence and metastasis (which include those tumors with an aggressive histopathologic subtype such as infiltrative, micronodular, morpheaform, and sclerosing). Risk factors for the development of BCC include exposure to ultraviolet radiation, a family history of BCC, and inherited or de novo mutations of the patched tumor-suppressor genes 1 and 2 (*PTCH1* and *PTCH2*) [1-8].

Melanoma and non-melanoma skin cancers (including squamous cell carcinoma and Merkel cell carcinoma) have invasive subtypes and in situ neoplasms [9-15]. Recently, in 2022, an in situ subtype of BCC was described [16]. Previously, these variants of BCC had been referred to as either a superficial BCC or a fibroepithelioma of the Pinkus type of BCC [1-8]. In summary, similar to other cutaneous neoplasms such as squamous cell carcinoma and melanoma, BCC also has an in situ subtype [16-21]. The nomenclature used by clinicians is of paramount importance. It enables healthcare professionals to efficiently communicate about their patients. The appropriate designation of a neoplasm can have substantial implications regarding its management and follow-up.

The National Cancer Institute has provided extensively clear guidelines regarding the definition of an in situ carcinoma and an invasive cancer [22,23]. With regards to a primary cutaneous neoplasm, an in situ carcinoma is characterized by a tumor with neoplastic cells that are restricted to the epidermis; strands of tumor cells contiguous with the overlying epidermis may extend into the underlying dermis [22]. In contrast, invasive skin cancer is characterized by a tumor with neoplastic cells that have originated from the epidermis and are independently present in the dermis in a non-contiguous manner with the overlying epidermis [23].

BCC is a cutaneous neoplasm [1-48]. Two of its pathologic variants have previously been inappropriately designated as invasive neoplasms. Specifically, the superficial type of BCC does not demonstrate a non-contiguous invasion of tumor cells from the epidermis into the dermis; it should be referred to as a BCC in situ [16-21]. In addition, the fibroepithelioma of Pinkus type of BCC shows an extension of tumor cell strands that originate and are contiguous with the epidermis into the dermis; this variant of BCC does not show independent tumor cell invasion in the dermis and also should be referred to as a BCC in situ [17].

The characteristics of nine individuals with cutaneous BCC in situ who we have identified and reported in the world literature are summarized in this review [16-21]. One of the cases involved a male with metastatic BCC who also developed two in situ BCCs while his metastatic disease was responding to treatment with the checkpoint inhibitor nivolumab. Genomic evaluation of the metastatic BCC of his liver and the in situ BCC of his skin suggested that the neoplasms were derived from different clones of cells [20,21].

## Review

Cancer: invasive or in situ

Neoplasms are either invasive or in situ. Invasive cancer, also called infiltrating cancer by the National Cancer Institute, is a tumor that grows into surrounding healthy tissues and has spread beyond the layer of tissue in which it developed [22]. In contrast to invasive cancer, the National Cancer Institute defines carcinoma in situ as a “condition in which abnormal cells that look similar to cancer cells under a microscope are found only in the place where they first formed and have not spread to nearby tissues” [23]. At some point, these cells may become cancerous and spread into nearby tissue.

Cutaneous carcinomas are invasive tumors in which the cancer cells that are present in the dermis are not contiguous with the overlying epidermis. In contrast, carcinomas in situ of the skin only contain neoplastic cells in the epidermis. However, in cutaneous carcinoma in situ, there may be an extension of the tumor cells that are contiguous with the overlying epidermis into the underlying dermis [16-21].

Basal cell epithelioma

In the 1940s, BCC - referred to as a basal cell epithelioma - was included in the myriads of benign neoplasms and malignant tumors classified as "epithelioma" [24,25]. In 1946, Elliot and Welton published a retrospective report of 1,742 patients with a total of 1,928 epitheliomas treated over a 22-year period from 1919 to 1941; only 381 of the 1928 cases had a histologic examination and they showed basal cell epithelioma (48.8%), squamous cell epithelioma (41.7%), or a combined basal and squamous cell epithelioma (9.4%) [24]. Subsequently, in 1949, Welton et al. published their clinical and histological data on 1,025 epithelioma lesions from 1,257 additional patients seen from 1941 to 1947; these included squamous cell epithelioma (such as benign seborrheic warts and malignant tumors, 30.3%), acanthoma (bordering malignancy, 3.4%), adnexal carcinoma (benign and malignant, 57.2%), basal cell epithelioma (2.1%), and transitional carcinomas (6.9%) [25].

Intraepidermal basal cell epithelioma

Borst, in 1904, introduced the concept of an intraepidermal basal cell epithelioma; 22 years later in 1926, Jadassohn reported a patient, cited Borst’s paper, and popularized this tumor. After that, it was commonly referred to as an intraepidermal basal cell epithelioma of Borst-Jadassohn; eventually, intercellular bridges were observed on the cells that composed the intraepidermal islands in the presumed intraepidermal basal cell epithelioma. Hence, the neoplasm was realized to represent a benign clonal seborrheic keratosis and not an in situ carcinoma of basaloid tumor cells [26,27].

Superficial basal cell epithelioma

Madsen described the superficial basal cell epithelioma as an incipient tumor in 1941 [28]. Other researchers have characterized the associated risk factors of basal cell epithelioma [29]. Eventually, the designation of carcinoma replaced the term epithelioma and the basal cell epithelioma came to be known as BCC; however, an in situ subtype of BCC was not established after the reclassification of the intraepidermal basal cell epithelioma.

Cutaneous BCC in situ

Based on the National Cancer Institute's definitions of an invasive cancer and an in situ carcinoma, it has been recently proposed in 2022 that the previously designated superficial BCC of the skin is cutaneous in situ (Table 1) [16-21]. Subsequently, in 2024, the subtypes of cutaneous BCC in situ were expanded to include not only a superficial variant (which is similar in pathology to what was referred to as a superficial BCC) but also a fibroepithelioma variant (which is similar in pathology to what was referred to as a fibroepithelioma of Pinkus) [17]. Later, in 2024, a case series of electron microscopy evaluation of BCCs demonstrated that the basal lamina in superficial BCC remained intact but showed breakage in both a nodular BCC and an infiltrative BCC; based on their observations, the investigators concluded that it would be appropriate to refer to the superficial BCC as a BCC in situ [18]. Also, in 2024, an editorial including a case report was published [19]. Finally, one of our patients with metastatic BCC had developed two new cutaneous BCC in situ while his metastatic disease was successfully responding to immunotherapy (complete remission) with the checkpoint inhibitor nivolumab [20,21].

**Table 1:** Summary of published studies of cutaneous basal cell carcinoma in situ BCC: basal cell carcinoma; CR: current report; PD-L1/2: programed death-ligand 1 and 2; Ref: reference

Author	Year	Comment	Ref
Cohen et al.	2024	Report of a patient with cutaneous BCC in situ with a review of the world literature on BCC in situ of the skin with emphasis on molecular studies	CR
Cohen	2024	Case report containing editorial stating that superficial BCC of the skin is a cutaneous BCC in situ	[19]
Kaur et al.	2024	Electron microscopy evidence that the tumor nodules from a patient with the non-invasive BCC in situ of the skin had an intact basal lamina in contrast to breakage in the continuity of the basal lamina of the invasive tumors of a patient with either a nodular or infiltrative subtypes of BCC	[18]
Cohen	2024	Two case reports of BCC in situ are presented; the paper also proposes two subtypes of in situ BCC: the superficial type and the fibroepithelioma type	[17]
Cohen	2022	Initial case series of cutaneous BCC in situ describing not only the clinical features but also the pathologic findings of the neoplasm in three men	[16]
Cohen et al. and Ikeda et al.	2017 and 2016	Retrospective diagnosis of two newly appearing BCCs in situ of the skin in a male patient with metastatic BCC to his brain, bone, liver, and soft tissue; the cutaneous BCCs in situ appeared while he was receiving the checkpoint inhibitor nivolumab and his metastatic disease had nearly completely resolved. Similar to other variants of BCC, genomic evaluation of his liver metastasis and his in situ cutaneous carcinoma demonstrated an aberration of the *PTCH1* gene; however, in contrast to his liver metastasis, the in situ carcinoma had a somewhat lower tumor mutational burden, lacked *PD-L1/2* amplification and had a distinct *PTCH1* mutation. These observations suggest that the in situ BCC of the skin and the metastatic basal cell cancer of his liver are derived from different clones of cells	[20, 21]

Cutaneous BCC in situ: epidemiology - age and gender

Nine patients (with ages ranging from 43 to 77 years, median: 62 years) with BCC in situ of the skin have been described in the world literature (Table 2) [16-21]. However, the cancers in these patients reflect a more general phenomenon, in that superficial BCCs may often be in fact in situ cancers. One of the men had two tumors. The individuals included seven men (with ages ranging from 43 to 69 years, median: 61 years) and two women (with ages ranging from 63 to 77 years, median: 70 years).

**Table 2:** Epidemiologic characteristics of patients with cutaneous basal cell carcinoma in situ ^a^Number of months that tumors were present before diagnosis BCC: basal cell carcinoma; CR: current report; FST: Fitzpatrick skin type (type 1 refers to individuals with skin that always burns and never tans); SCC: squamous cell carcinoma

Case	Age	Race	Gender	FST	Skin cancer history	Duration^a^	Symptoms	References
1	43 years	Caucasian	Male	1	None	6 months	None	[16], C1
2	51 years	Caucasian	Male	1	BCC	<12 months	None	CR
3	58 years	Caucasian	Male	1	BCC	<3 months	None	[20,21]
					BCC	<3 months	None	[20,21]
4	61 years	Caucasian	Male	1	None	Not stated	None	[17], C2
5	62 years	Caucasian	Male	1	Not stated	Not stated	None	[19]
6	63 years	Caucasian	Male	1	BCC, SCC	4 months	None	[16], C2
7	63 years	Caucasian	Male	1	None	Not stated	None	[17], C1
8	69 years	Caucasian	Male	1	None	Not stated	None	[16], C3
9	77 years	Caucasian	Female	1	Not stated	Not stated	Not stated	[18]

Cutaneous BCC in situ: epidemiology - race and skin type

All the patients were Caucasian (Table 2) [16-21]. In addition, all the patients were Fitzpatrick skin type 1. Therefore, these were all individuals with skin that would always burn after exposure to the sun but would never tan [16-21].

Cutaneous BCC in situ: epidemiology - skin cancer history

Three of the men had a history of prior skin cancer including not only BCC (three men) but also squamous cell carcinoma (one man) (Table 2) [16-21]. None of the patients’ in situ carcinoma were symptomatic. The neoplasm had been present before diagnosis, with the duration ranging from less than three months to less than one year; the median duration was four months [16-21].

Cutaneous BCC in situ: location

The in situ carcinoma was most commonly located on either the abdomen (three tumors) or the upper extremity (three tumors); it was also found on the back (two tumors) and chest (two tumors) (Table 3) [16-21]. At diagnosis, the neoplasm ranged in diameter from 6 x 6 millimeters to 5 x 4 centimeters; three tumors had the greatest diameter of 5 centimeters, two tumors were smaller than 1 centimeter, and the length of three tumors was between 1-5 centimeters [16-21].

**Table 3:** Clinical features of patients with cutaneous basal cell carcinoma in situ ^a^Correlation of the clinical presentation and pathology changes established the diagnosis of cutaneous BCC in situ of the superficial type. ^b^Correlation of the clinical presentation and pathology changes established the diagnosis of cutaneous BCC in situ of the fibroepithelioma type. BCC: basal cell carcinoma; CR: current report; EdC: electrodessication and curettage; Ex: excision; F-up: follow-up (months no recurrence); IC: imiquimod cream (5%); NS: not stated; Refs: reference; SEx: shave excision during biopsy; Tx: treatment

Case	Site	Size	Morphology	Tx	F-up	Ref
1	Right upper abdomen	5 x 4 x 4 cm	Erythematous, triangular-shaped, plaque^a^	Ex	15	[16], C1
2	Right dorsal hand	2 x 1 cm	Erythematous, scaly, plaque with raised peripheral border^a^	Ex	42	CR
3	Left anterior shoulder (adjacent to chest)	0.6 x 0.6 cm	Erythematous plaque^a^	EdC	8	[20,21]
	Left chest	0.8 x 0.6 cm	Erythematous plaque^a^	EdC	8	[20,21]
4	Left mid back	3 x 2 cm	Smooth, flesh-colored, exophytic nodule^b^	SEx	24	[17], C2
5	Right mid-upper arm	NS	Erythematous, scaly, plaque^a^	Ex	NS	[19]
6	Right lateral lower abdomen	2 x 2 cm	Erythematous, scaly, plaque with peripheral nodules^a^	IC	21	[16], C2
7	Left lower abdomen	5 x 3 cm	Erythematous, scaly, plaque^a^	Ex	48	[17], C1
8	Left lower back	4 x 2.5 cm	Erythematous, focally crusted, plaque with peripheral nodules^a^	Ex	24	[16], C3
9	Forearm	NS	Erythematous, scaly, plaque^a^	NS	NS	[18]

Cutaneous BCC in situ: clinical features

**Figure 1:**
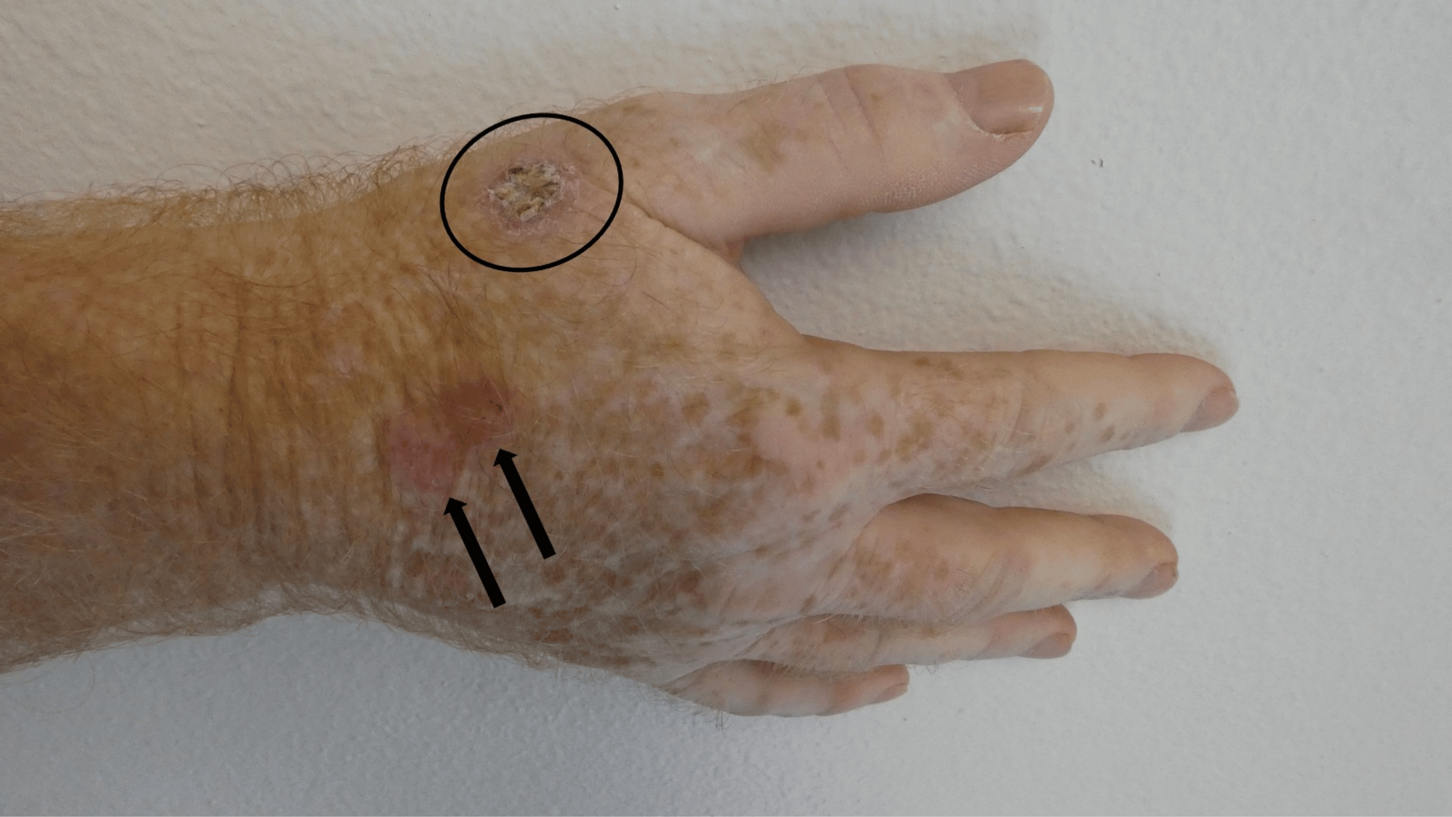
Clinical presentation of a basal cell carcinoma (BCC) in situ of the skin on the right dorsal hand A 51-year-old male with a history of actinic keratoses and basal cell carcinomas (BCCs) presented with a painless, non-pruritic lesion that had been present for less than a year and had progressively increased in size. The BCC in situ appeared as 2 x 1-centimeter scaly erythematous plaques with a raised peripheral edge on the right dorsal hand proximal to the thumb (black oval); a shave biopsy was performed. The two erythematous patches (black arrows) are the sites of actinic keratoses that were treated with liquid nitrogen cryotherapy. In addition, features of vitiligo are demonstrated by the depigmentation of the distal dorsal hand and the fingers.

The cutaneous BCC in situ presented with clinical features of those previously described in superficial BCC in nine of the tumors [1-7]. The neoplasm appeared as an erythematous plaque (Table 3) [16-21]. Five of the tumors were scaly and one of the tumors was crusted (Figure 1) [16-21]. The peripheral border was raised in two of the tumors. One of the tumors on the back presented as a smooth, flesh-colored exophytic nodule; this morphologic presentation was similar to what was previously described for fibroepithelioma of Pinkus tumors [8,17].

Cutaneous BCC in situ: diagnosis

Challenges exist not only in clinically diagnosing early stage BCC but also in distinguishing BCC in situ from other precancerous or cancerous lesions such as actinic keratosis and squamous cell carcinoma in situ. The diagnosis of BCC in situ may be considered based on the appearance of the clinical lesion. Dermoscopy is a noninvasive technique; it may be used to aid in the evaluation of the skin lesion [1,47,48]. The findings on dermoscopy of a BCC in situ would be expected to be those previously observed and described for superficial BCC. Some of these include short fine telangiectasias, shiny white-red structureless background areas, multiple small erosions, and areas of leaf-like pigmentation [1,47,48]. However retrospective studies have described dermatoscopic features of superficial BCC that are either unusual or mimic those of infiltrative or morpheaform BCC [47].

The diagnostic accuracy of dermoscopy can be improved by incorporating other novel in vivo diagnostic tools: optical coherence tomography, reflectance confocal microscopy, high-resolution ultrasonography, Raman spectroscopy, and terahertz pulse imaging. The inclusion of these modalities also enables the evaluation of tumor depth and lateral margins without the use of invasive techniques. Although the diagnosis of BCC in situ may be suspected based on the clinical presentation and supported by dermoscopic findings, it is reasonable to perform a microscopic assessment of a biopsy taken from the lesion to confirm the diagnosis and exclude the possibility of a BCC with mixed histology [1,47,48].

Cutaneous BCC in situ: treatment

Treatment included excision of six tumors; the fibroepithelioma-like in situ carcinoma had been completely removed during the shave biopsy performed for diagnosis (Table 3) [16-21]. The two in situ BCC in the male patient with metastatic BCC were treated by electrodessication and curettage. One tumor was treated topically using 5% imiquimod cream that was applied to the in situ carcinoma and surrounding skin for five consecutive nights each week for six weeks [16-21].

Cutaneous BCC in situ: follow-up

Follow-up ranged from eight to 48 months (Table 3) [16-21]. The median duration of follow-up was nearly two years. There was no recurrence of the in situ carcinomas [16-21].

Cutaneous BCC in situ: pathology

**Figure 2:**
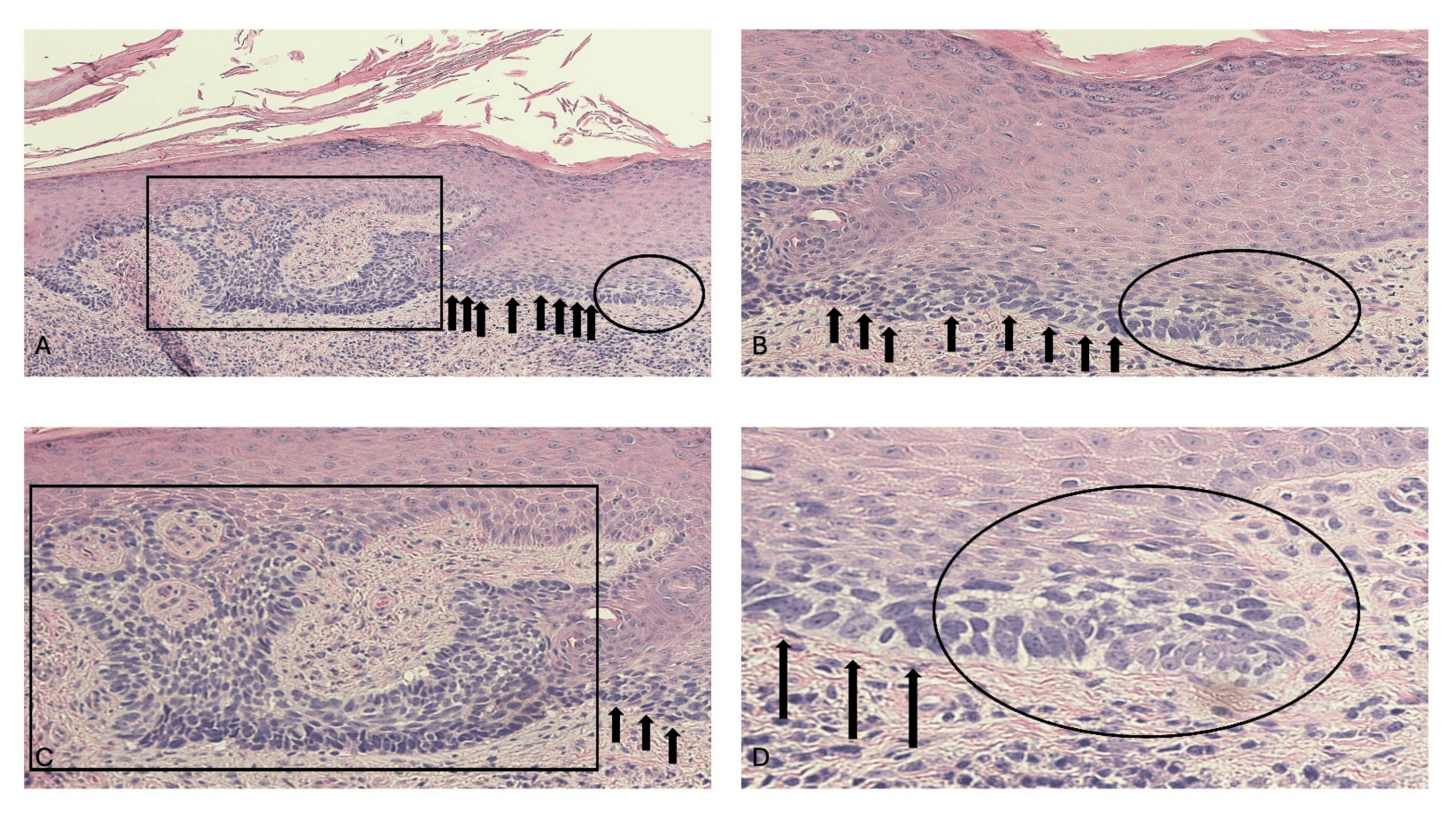
Microscopic features of a cutaneous basal cell carcinoma (BCC) in situ on the right dorsal hand of a 51-year-old male Distant (A) and closer (B, C, and D) views demonstrate the pathologic changes the right dorsal hand BCC in situ. There is compact orthokeratosis (demonstrated by thickening of the keratin layer in the stratum corneum with preserved keratinocyte maturation and without retained nuclei) overlying the epidermis (which is the outermost layer of the skin which is composed of several strata from the most superficial to the deepest including the stratum corneum, stratum lucidum, stratum granulosum, stratum spinosum, and stratum basale); this finding correlates with the scaling observed clinically. Superficial buds of basaloid tumor cells that are contiguous with the epidermis extend into the papillary dermis and anastomose (shown within the black rectangle); also, there is an aggregate of tumor cells present in the lower layers of the epidermis (within the oval). At the periphery of the anastomosing aggregates of in situ BCC, there is peripheral palisading of the tumor keratinocytes. In addition, there is a confluent proliferation of atypical cells along the basal layer of the epidermis (black arrows). Importantly, there were no basaloid tumor cells that were not contiguous with the epidermis, in the dermis. In the papillary dermis there is a lymphocytic inflammatory infiltrate that is predominantly around the blood vessels, but also diffusely present between the collagen bundles. Correlation of the clinical morphology and the pathologic findings established a diagnosis of cutaneous BCC in situ. The residual tumor was excised and there was no recurrence of the in situ carcinoma after three-and-a-half years (hematoxylin and eosin: A, x10; B, x20; C, x 20; D, x 40).

Microscopic evaluation of the cutaneous BCC in situ demonstrated two pathologic subtypes. The first subtype had pathologic features like those previously described in superficial BCCs (Figure 2) [1-7]. Basaloid tumor cells were present along the basal layer of the epidermis; in addition, the tumor cells not only replaced the lower layers of the epidermis but in some circumstances nearly filled the epidermis. In addition, in some of the tumors, the aggregates of basaloid tumor cells not only expanded the epidermis, or contiguously extended from the epidermis into the underlying papillary dermis, or both. Of note, there was no invasion of tumor cells that were not attached to the overlying epidermis into the dermis [16-21].

The second subtype had pathologic features like those previously described in the fibroepithelioma of Pinkus tumors [8]. Contiguous strands of basaloid tumor cells extended from the epidermis into the underlying dermis. There was no invasion of tumor cells that were not attached to the overlying epidermis into the dermis [17].

BCC with mixed histology

A pathologic subtype of BCC includes BCC with mixed histology. These tumors contain one or more subtypes of BCC. They can include two or more nonaggressive pathologic subtypes, one or more nonaggressive and aggressive pathologic subtypes, or two or more aggressive pathologic subtypes. Previously patients have been observed to have superficial BCC in the epidermis and an aggressive subtype, such as infiltrative BCC in the underlying dermis; currently, this tumor would be interpreted as a BCC in situ overlying an invasive BCC in the dermis. All the patients in whom cutaneous BCC in situ has been reported only had tumors that contained an in situ component. When the possibility of a BCC with mixed histology is entertained, the clinician might consider performing a deeper punch biopsy instead of a more superficial shave biopsy to assess the lesion [30-33].

Cutaneous BCC in situ: electron microscopy

Electron microscopy studies have demonstrated the differences between BCC in situ (referred to as superficial BCC in the studies performed) and other pathologic subtypes of BCC [18,34-37]. Kalliginen et al. in 1984 compared six nonfibrosing BCC and seven fibrosing BCC; all the nonfibrosing BCC had continuous and distinct basement membranes [34]. However, three of the fibrosing BCC had discontinuous basement membranes and the remaining four fibrosing had a basement membrane that was continuous but indistinct [34].

Three years later, in 1987, Barsky et al. evaluated 30 BCC cases: 15 cases of nonaggressive BCC (ten nodular-ulcerative BCC and five superficial BCC) and 15 cases of aggressive desmoplastic BCC [35]. The basal lamina was completely present in all 15 cases of nonaggressive BCC; it was either thickened (six cases), reduplicated (five cases), or normal (four cases). However, the basal lamina was absent --either completely (five cases) or 50-75% absent (10 cases)--in all the desmoplastic BCC [35].

In 1998, 11 years later, Hayakawa et al. studied 28 BCC cases: 11 tumors that had indolent growth and were nonaggressive (superficial and circumscribed) BCC and 17 aggressive (infiltrative) BCC [36]. In contrast to Barsky et al.’s observation, Hayakawa et al. observed a markedly diminished thickness of the basement around the nonaggressive BCCs; they speculated that this change might promote stromal-tumor signaling in these tumors. They also noted that the nonaggressive BCC had a reduced concentration of hemidesmosomes, abnormalities of tonofilament-desmosomal association, and disorder of the tonofibril shell [36].

Crowson, who was one of Hayakawa’s coinvestigators, included electron micrographs in a review article on BCC eight years later in 2006 [37]. The illustration of the BCC with infiltrative growth demonstrated no delimiting zone of the basement membrane. However, the legend of the nodular BCC stated that “the nodular BCC is characterized by rounded nests of neoplastic basaloid cells that are delimited by an almost continuous basal lamina” [37].

Recently, in 2024, Kaur et al. meticulously examined the electron micrographs of a superficial BCC, a nodular BCC, and an infiltrative BCC [18]. Similar to the aggressive tumors studied by Kalliginen et al., Barsky et al., and Crowson, there was breakage in the basal lamina of the tumor nests of the infiltrative BCC. However, in contrast to Barsky et al., and like Crowson, the nodular BCC showed breakage in the basal lamina. Finally, also like the nonfibrosing tumors evaluated by Kalliginen et al. and the nonaggressive BCC studied by Barsky et al., the basal lamina was intact around the tumor cells of the superficial BCC [18].

Cutaneous BCC in situ: molecular evaluation

Molecular evaluation of cutaneous BCC in situ has been performed in a male patient who had metastatic BCC to his brain, bone, liver, and lungs. The in situ BCC of the skin presented while he was being treated with the checkpoint inhibitor nivolumab and had achieved over 95% regression on imaging studies of his metastatic disease. Genomic analysis was performed of both the metastatic BCC of the liver and the BCC in situ of the skin (Table 4) [20,21].

**Table 4:** Genomic aberrations of a patient’s cutaneous basal cell carcinoma in situ and his liver basal cell carcinoma metastasis ^a^In 2016, the left anterior shoulder BCC in situ had next-generation sequencing performed. ^b^In 2015, the liver biopsy was repeated; the diagnosis of BCC was confirmed; and hybrid capture-based next-generation sequencing (236 genes) was performed. ^c^The possibility of a germline mutation is raised by the unifying molecular aberration in the *CTNNA1* gene since a coincidental mutation in the same locus (*CTNNA1 R383H*) of the same gene in both tumors would be unlikely. BCC: basal cell carcinoma

Genomic alteration	Cutaneous BCC in situ (2016)^a^	BCC liver metastasis (2015)^b^
AXIN1 T601	Yes	No
BAP1 K368*	Yes	No
CARD11 E756K	Yes	No
CDKN1A R140Q	No	Yes
CDKN2A p16INK4a P81L	No	Yes
CTNNA1 R383H^c^	Yes	Yes
FLT1 E487K	No	Yes
*JAK2* amplification	No	Yes
KDR R1032Q	Yes	No
*LRP1B* splice site *9121-1G>A*	No	Yes
LRP1B W2334*	No	Yes
*MLL2* splice site *4132-1G>A*	No	Yes
NOTCH1 W287*	No	Yes
PDGFRA E459K	No	Yes
*PD-L1 (CD274) *amplification	No	Yes
*PD-L2 (PDCD1LG2) *amplification	No	Yes
PIK3R2 Q412*	No	Yes
PTCH1 Q1366*	No	Yes
PTCH1 S181*, splice site *584+1G>A*	Yes	No
PTCH1 W197*	No	Yes
SLIT2 K325*	No	Yes
SMARCA4 Q1166*	No	Yes
SPEN R1854Q	Yes	No
TERT promoter-139_-138CC>TT	No	Yes
TP53 E285K	Yes	No
TP53 P278S	No	Yes
Total characterized alterations	8	19
Tumor mutation burden (mutations/megabase)	45	103

Cutaneous BCC in situ: molecular evaluation - clinical features

In 2012, at age 54 years, the patient initially developed a BCC on his left posterior shoulder; this tumor was excised. However, the left posterior shoulder cutaneous BCC recurred and was excised; the postoperative wound was also treated with radiotherapy since the margins of the excision were positive for the tumor. Within two years, in 2014, he presented with back pain; the workup not only showed recurrence of the BCC presenting as a left posterior shoulder soft tissue tumor but also BCC metastases to his axial skeleton, liver, and lungs; the cancer diagnosis of BCC was confirmed by biopsy of the soft tissue, bone, and liver.

He was treated with vismodegib in 2014; however, within three months, he developed brain metastases. Subsequent treatments included stereotactic radiosurgery (which was successful), cisplatin with paclitaxel (during which he developed progressive bone and liver metastases), sonidegib with buparlisib (during which he continued to develop metastatic liver disease), and vismodegib with paclitaxel (which demonstrated no response to therapy). A liver biopsy was repeated in 2015, again confirming the diagnosis of BCC; hybrid capture-based next-generation sequencing (236 genes) was performed (Table 4) [20,21]. Successful management of his metastatic disease rapidly occurred with tumor shrinkage to near-complete remission after starting treatment with the checkpoint inhibitor nivolumab [20,21].

In 2016, two new BCCs in situ appeared on the left anterior shoulder and the left clavicle of a 58-year-old man, while he was being treated with nivolumab and had achieved near-complete remission of his metastatic BCC (which had involved his brain, bone, liver, and lungs). Next-generation sequencing of the left anterior shoulder BCC in situ was performed (Table 4) [20,21]. Both cutaneous in situ BCCs were treated with electrodessication and curettage. In 2017, follow-up did not show any recurrence of the skin BCCs in situ; he was still receiving nivolumab and had achieved over 95% regression on imaging of his metastatic BCC [20,21].

Cutaneous BCC in situ: molecular evaluation - genomic analysis

The metastatic BCC in his liver was tested using hybrid capture-based next-generation sequencing (236 genes). A tumor mutational burden of 103 mutations per megabase (where 19 mutations per megabase are considered a high tumor mutation burden) and 19 genomic alterations including amplification of programmed death-ligand 1 (*PD-L1*), programmed death-ligand 2 (*PD-L2*) and Janus kinase 2 (*JAK2*) by sequencing; indeed, his metastatic disease demonstrated an exceptional response to anti-programmed death 1 (PD1) therapy [20,21].

Next-generation sequencing of the in situ BCC from his left shoulder showed a lower tumor mutational burden of 45 mutations per megabase and only eight genomic alterations. In contrast to the metastatic BCC in his liver, the primary BCC in situ of the skin did not exhibit amplification of *PD-L1, PD-L2*, or *JAK2*. The new skin neoplasms were successfully treated locally by electrodessication and curettage [20,21]. A common genomic alteration of *CTNNA1 R383H* was observed in both the metastatic BCC in the liver and the in situ BCC of the skin. It would be unlikely for a coincidental mutation in the same locus of the same gene to occur in both tumors. Therefore, the possibility of a germline mutation in the *CTNNA1* gene is raised based on the unifying molecular aberration [20,21,38].

In summary, in a man with metastatic BCC, aberration of the *PTCH1* gene was observed during the genomic evaluation of his liver metastasis and cutaneous BCC in situ that developed while he was being treated with nivolumab. In contrast to the liver BCC metastasis, the cutaneous in situ BCC had a lower tumor mutational burden, lacked *PD-L1/2* amplification, and had a distinct *PTCH1* mutation. Therefore, it is reasonable to postulate that the in situ BCC of his skin and the metastatic BCC of his liver were derived from different clones of cells [20,21].

Treatment of BCC with aggressive pathologic subtypes

There are several potential approaches to the treatment of BCC. Surgical excision, with methods such as Mohs micrographic surgery or without microscopic evaluation of the margins during the procedure, is most commonly used for BCC with aggressive pathologic subtypes. In some patients, radiation therapy may be appropriate. Hedgehog pathway inhibitors (such as vismodegib and sonidegib) and anti-PD-1 immunotherapy (including U.S. Food and Drug Administration-approved therapy using cemiplimab, or experimental therapy using either pembrolizumab or nivolumab in our patient) may be utilized for locally advanced or metastatic BCC [1-6,39,40].

Treatment of BCC with nonaggressive pathologic subtypes

However, in patients with nonaggressive subtypes of BCC, additional management options are available. All these treatment alternatives would also be appropriate for cutaneous BCC in situ. Some of the possibilities include destructive therapies: cryosurgery (a local treatment that uses liquid nitrogen to provide extreme cold to destroy the cancer cells), electrodessication, and curettage (in which the sequence of using a curette to scrape and remove the tumor before the area being treated with an electrode to destroy any remaining cancer cells is repeated once or twice during the treatment session), and laser ablation using either a carbon dioxide laser or a neodymium-doped yttrium aluminum garnet (ND:YAG) laser (in which the cancer cells are destroyed by irradiating the tumor with a high-intensity beam of light). Other therapeutic modalities are photodynamic therapy (in which drugs that are activated by light, such as either the photosensitizer 5-aminolevulinic acid or methyl aminolevulinate is topically applied to the tumor and light is directed toward the sensitized tumor--using either a light-emitting diode lamp, a halogen lamp or a laser-to destroy the cancer cells), and topical therapies (such as imiquimod and 5-fluorouracil) [1-5,39]. 

BCC and BCC in situ in elderly patients: potential management

In older patients with a BCC, it might be reasonable to evaluate life expectancy when determining the approach to treatment; in these individuals, the frailty, the functional status, and the potential comorbidities of the therapy should be assessed. In elderly patients who have a limited life expectancy, watchful waiting and active surveillance might be considered a reasonable consideration for the management of an in situ BCC of the skin [41-46].

Treatment of BCC in situ: future considerations

Cutaneous BCC in situ is a nonaggressive pathologic subtype of BCC. The 10 in situ carcinomas in the patients in this review were successfully managed: excision (six tumors), electrodessication and curettage (two tumors), and 5% imiquimod cream (one tumor). Based on their pathology, a reasonable approach to the management of BCC in situ could include modalities that have been successfully used to treat BCCs with nonaggressive pathology subtypes. In addition, in the appropriate setting involving elderly patients whose life expectancy is limited, watchful waiting and active surveillance may be considered.

Limitations of the review

This review is based on a small number of tumors (10), which may limit the generalizability of the findings and conclusions drawn; also the cited papers demonstrate the heterogeneity of study designs and methodologies. The article also lacks statistical analysis and quantitative data to support the prevalence and significance of the findings. In addition, the selection of cases and literature might introduce certain biases, as the review does not discuss the criteria for including or excluding certain studies. And, the genomic data from only one individual are discussed, which may not fully represent the variability found in different patients with BCC in situ.

Summary

Cutaneous BCC in situ is a recently recognized subtype of BCC. With regards to cancers of the skin, invasion designates that individual or aggregates of tumor cells that are not contiguous with the overlying epidermis can be found in the underlying dermis. In contrast, in an in situ skin neoplasm, the abnormal cells are confined to the epidermis; however, there may be an extension of the cancer cells, that are contiguous with the overlying epidermis into the underlying dermis. BCC in situ of the skin was previously referred to as a superficial BCC.

A review of the world literature has identified 10 cutaneous BCCs in situ that have been described in nine patients. However, these cases reflect a more widespread phenomenon. The neoplasm typically presents as an asymptomatic red plaque on the abdomen, upper extremity, back, and chest; pathologic changes frequently show confluent tumor cells along the basal layer of the epidermis or superficial aggregates of neoplastic cells that are contiguous with the overlying epidermis and extend into the underlying papillary dermis. The fibroepithelioma subtype of cutaneous BCC in situ morphologically presents as a smooth, flesh-colored exophytic nodule and microscopically demonstrates cutaneous strands of basaloid tumor cells extending from the epidermis into the dermis. Successful treatment of the in situ BCCs includes excision, electrodessication and curettage, and topical application of imiquimod cream.

Electron microscopy studies have recently demonstrated objective differences between cutaneous BCC in situ as compared to invasive subtypes of BCC such as nodular BCC and infiltrative BCC. The in situ BCC of the skin has an intact basal lamina. In contrast, the invasive types of BCC both show breakage in the basal lamina.

The molecular pathogenesis of BCC in situ of the skin is suspected to be similar to that of other subtypes of BCC and results from a genomic aberration in the *PTCH1* gene. Next-generation sequencing of cutaneous BCC in situ has been performed; the patient had metastatic BCC, originating from a soft tissue tumor with liver metastases. His metastatic cancer was demonstrating an ongoing response to an anti-PD1 checkpoint inhibitor (nivolumab); however, two new BCCs in situ of the skin developed. In contrast to the very high mutational burden of his metastatic liver BCC (103 mutations per megabase), his in situ BCC had a lower tumor mutational burden (45 mutations per megabase). In addition, the metastatic tumor showed *PD-L1/2* amplification while the in situ tumor did not. Finally, both primary and metastatic tumors showed *PTCH1* mutations, though the site of the mutation differed.

There are important therapeutic implications regarding the identification of in situ BCC of the skin. Traditionally, the treatment of BCCs that have aggressive subtypes, are locally advanced, or are metastatic requires either surgery, radiotherapy, Hedgehog pathway inhibitors, and/or anti-PD-1 immunotherapy. However, for cutaneous BCC in situ, locally destructive therapies, photodynamic therapy, or topical therapy may be considered. The fibroepithelioma variant of BCC in situ is often completely removed at the time of biopsy or with subsequent conservative surgical re-excision of the site. In addition, in elderly patients who have a limited life expectancy, active surveillance and watchful waiting may be a reasonable approach to the management of a BCC in situ of the skin.

## Conclusions

Like other cutaneous neoplasms such as squamous cell carcinoma and melanoma, BCC also has an in situ subtype. BCC in situ of the skin has a distinctive clinical presentation and pathologic features. Genomic evaluation has been performed in neoplasms from one individual with metastatic BCC; similar to other variants of BCC, an aberration of the *PTCH1* gene was observed. In contrast to his liver metastasis, the in situ carcinoma had a somewhat lower tumor mutational burden, lacked *PD-L1/2* amplification, and had a distinct *PTCH1* mutation, suggesting that the in situ BCC of his skin and the metastatic BCC of his liver were derived from different clones of cells. Tumor-focused treatment of cutaneous BCC in situ may be appropriate for the management of this non-invasive neoplasm; also, for elderly patients who have a limited life expectancy, watchful waiting with active surveillance of their BCC in situ of the skin might be considered.
